# 1369. Effect of COVID-19 on Antibiotic Consumption: An Analysis of the COVID-19 Community Research Partnership

**DOI:** 10.1093/ofid/ofad500.1206

**Published:** 2023-11-27

**Authors:** Olivia Randazza, Michael E DeWitt, John C Williamson

**Affiliations:** Atrium Health Wake Forest Baptist, Winston-Salem, North Carolina; Atrium Wake Forest Baptist Health/ Wake Forest University School of Medicine, Winston-Salem, North Carolina; Atrium Health Wake Forest Baptist, Winston-Salem, North Carolina

## Abstract

**Background:**

The COVID-19 Community Research Partnership (CCRP) is a multisite healthcare system-based study of the COVID pandemic. Community residents in this study provided consent for electronic health record (EHR) access allowing for analysis of antibiotic prescribing.

**Methods:**

We conducted a retrospective, self-controlled cohort study to determine antibiotic consumption in the CCRP pre-COVID (Jan 2018 - Dec 2019) and post-COVID (Jan 2020 - Jan 2022). Participants were ≥18 years with an EHR record in the pre-COVID period. The primary outcome was the number of antibiotic prescriptions (AP) per 1000 patient care encounters in each time period. Secondary outcomes included the AP location of care, analysis of antibiotic classes, and COVID attributable antibiotic consumption, defined as an AP written ≤14 days before or after an EHR documented or self-reported positive SARS-CoV-2 test or COVID diagnosis.

**Results:**

34,234 participants were included representing 2,775,711 encounters. Median age was 53 years with 70% female and 88% white. 41% lived in a rural or suburban area. There were 26,121 APs among 25,433 participants. Mean rate of AP per 1000 encounters was 12.1 and 7.6 pre vs post-COVID (p< 0.001). A decline in AP occurred at onset of the pandemic persisting through the post-COVID period (Fig 1). The prescribing rate for all antibiotics, except linezolid, decreased during the pandemic (p< 0.001, Fig 2). The largest declines were seen with antibiotics typically prescribed for respiratory illnesses. The decline in antibiotic consumption was driven by outpatient encounters (Fig 3). 6,341 participants were diagnosed with COVID and 333 APs were COVID attributable. These participants were more likely to receive a macrolide (45% vs. 22%, p< 0.001), live in a rural area (31% vs. 20%, p< 0.001), and receive care in an emergency department (12% vs. 6.1%, p< 0.001).

**Figure 1.**

**Figure:**
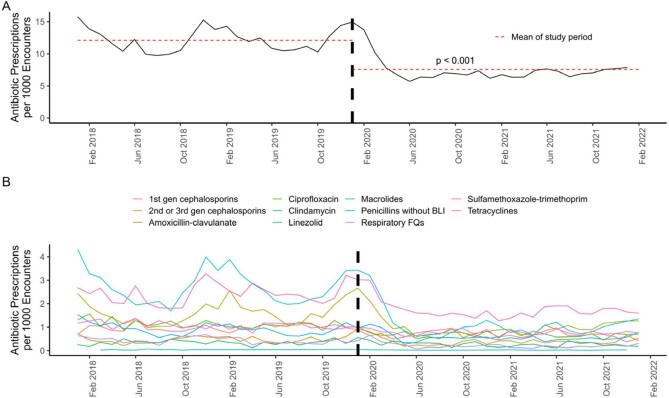

Antibiotic Prescriptions During The Study Time Period

**Figure 2.**

**Figure:**
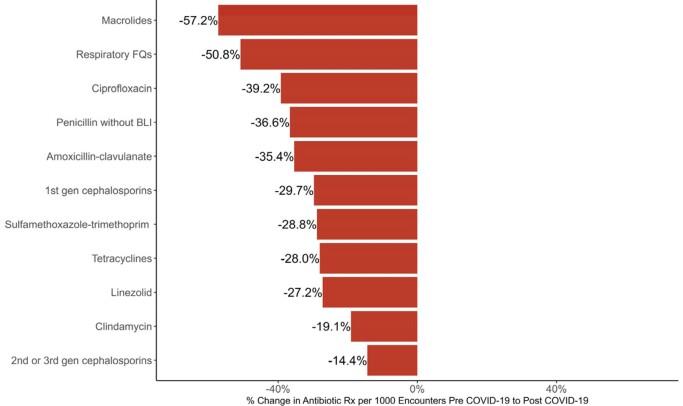

Analysis Of Antibiotic Classes

**Figure 3.**

**Figure:**
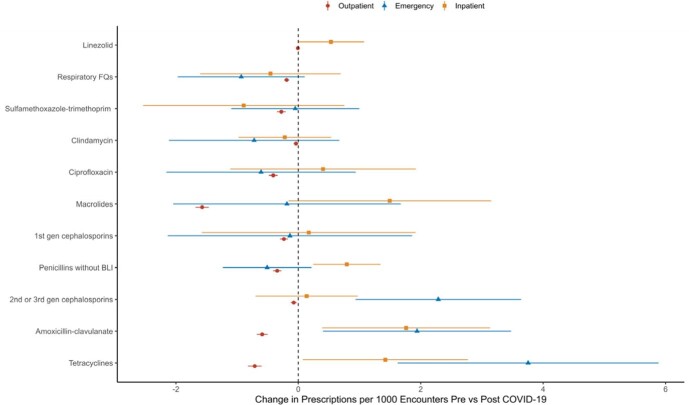

Location of Antibiotic Prescribing

**Conclusion:**

The COVID pandemic was associated with a broad decline in antibiotic consumption. These results may be due to declines in other respiratory viruses, anchoring bias for COVID during the pandemic, improved antibiotic stewardship practices, or social isolation. Antibiotics for respiratory illnesses declined the most, suggesting this may be a target for outpatient stewardship efforts.

**Disclosures:**

**All Authors**: No reported disclosures

